# Exploring biological response profiles to amyloid antibodies in clinical practice using Quantitative Systems Pharmacology

**DOI:** 10.1002/alz.087647

**Published:** 2025-01-09

**Authors:** Athena Grant, Shaina M Short, Hugo Geerts

**Affiliations:** ^1^ Certara UK Limited, Sheffield, S1 2BJ United Kingdom; ^2^ Certara, Park City, UT USA; ^3^ Certara SimCyp, Berwyn, PA USA

## Abstract

**Background:**

With the approval of several anti‐amyloid antibodies and a robust pipeline of new amyloid‐based therapies, attention turns towards questions related to real‐world clinical practice. Here we explore the impact of several biological pathways on the amyloid biomarker response of AD patients using a Quantitative Systems Pharmacology (QSP) approach with the ultimate objective to find measurable biomarkers for responder identification.

**Method:**

Using a well‐validated QSP biophysically realistic model of amyloid aggregation, we performed sensitivity analysis to identify key drivers of amyloid biomarkers both in a longitudinal observational context and after treatment with specific amyloid antibodies. One Factor at a Time (OFAT), Pareto plots and Big Data classification algorithms in large virtual patient trials were used.

**Result:**

Not unexpectedly, in observational studies the major driver of amyloid aggregation (age of decrease in CSF Ab42 and maximal SUVR readout) included parameters of the amyloid aggregation, notably the exponential weighting factor of the aggregate order on the backward (monomer dissociation) rate constants. Smaller contributions of the forward rate constants, synthesis of APP and microglia‐mediated clearance of amyloid species were found.

The same parameter was the major driver of SUVR response in trials with donanemab (dona) versus aducanumab (adu) and lecanemab (leca) with opposite effects on oligomers and protofibrils reduction. In contrast, parameters associated with secondary nucleation and flow from interstitial fluid (ISF) into cerebrospinal fluid (CSF) were driving ARIA‐E lability with opposing directions between adu versus dona and leca versus dona. The contribution of the different processes to the biomarker responses of the three antibodies were smaller in non‐APOE4 patients, except for the ARIA‐E liability where they were more pronounced.

**Conclusion:**

This approach can be used with the pharmacology of different therapies (including novel amyloid antibodies, gamma‐secretase modulators and oligomerisation inhibitors), to identify pathways characterizing individual patient responses.

